# Voluntary vaccination may not stop monkeypox outbreak: A game-theoretic model

**DOI:** 10.1371/journal.pntd.0010970

**Published:** 2022-12-14

**Authors:** Ian B. Augsburger, Grace K. Galanthay, Jacob H. Tarosky, Jan Rychtář, Dewey Taylor

**Affiliations:** 1 Department of Applied Mathematics and Statistics, Johns Hopkins University, Baltimore, Maryland, United States of America; 2 Department of Mathematics and Computer Science, College of the Holy Cross, Worcester, Massachusetts, United States of America; 3 Department of Mathematical Sciences, High Point University, High Point, North Carolina, United States of America; 4 Department of Mathematics and Applied Mathematics, Virginia Commonwealth University, Richmond, Virginia, United States of America; McGill University Faculty of Medicine and Health Sciences, CANADA

## Abstract

Monkeypox (MPX) is a viral zoonotic disease that was endemic to Central and West Africa. However, during the first half of 2022, MPX spread to almost 60 countries all over the world. Smallpox vaccines are about 85% effective in preventing MPX infections. Our objective is to determine whether the vaccines should be mandated or whether voluntary use of the vaccine could be enough to stop the MPX outbreak. We incorporate a standard SVEIR compartmental model of MPX transmission into a game-theoretical framework. We study a vaccination game in which individuals decide whether or not to vaccinate by assessing their benefits and costs. We solve the game for Nash equilibria, i.e., the vaccination rates the individuals would likely adopt without any outside intervention. We show that, without vaccination, MPX can become endemic in previously non-endemic regions, including the United States. We also show that to “not vaccinate” is often an optimal solution from the individual’s perspective. Moreover, we demonstrate that, for some parameter values, there are multiple equilibria of the vaccination game, and they exhibit a backward bifurcation. Thus, without centrally mandated minimal vaccination rates, the population could easily revert to no vaccination scenario.

## Author summary

Monkeypox (MPX) is a viral disease that recently spread to almost 60 countries all over the world. Our main goal is to determine whether the smallpox vaccines, which are about 85% effective against MPX, should be mandated or whether voluntary use of the vaccine could be enough to stop the MPX outbreak. We study a vaccination game in which individuals decide whether or not to vaccinate by assessing their benefits and costs. We show that, without vaccination, MPX can become endemic in previously non-endemic regions, including the United States. We also show that to “not vaccinate” is often an optimal solution from the individual’s perspective. Moreover, we demonstrate that, for some parameter values, there are multiple solutions of the vaccination game and without centrally mandated minimal vaccination rates, the population could easily revert to no vaccination scenario.

## 1 Introduction

### 1.1 Monkeypox

Monkeypox (MPX) is a viral zoonotic disease endemic to Central and West Africa [1]. The MPX cases suffer from mild symptoms such as headaches, fevers, rashes, lesions in their mouth and on their body [2], although there may be other and potentially severe complications such as blindness [3] or death [4]; see [5] for a comprehensive review.

It has recently garnered much public attention due to its 2022 outbreaks. From January 1 to July 4, 2022, 6027 laboratory confirmed MPX cases were reported to WHO from 59 countries, most of which are considered non-endemic to MPX [6]. Men who have sex with men (MSM) with new or multiple partners are amongst the most affected [7].

MPX is caused by a virus similar to smallpox virus and the smallpox vaccines provide about 85% protection from MPX [8]. For the current outbreak, [9] recommends (a) post-exposure prophylaxis (PEP) with an appropriate second- or third-generation vaccine for contacts of cases, and (b) pre-exposure prophylaxis (PrEP) for people at risk. However, as the MPX epidemic continues to unfold, there are calls to use the smallpox vaccine as PrEP in MSM at high risk of monkeypox virus exposure as it may also reduce transmission into the general population [7].

### 1.2 Mathematical models of MPX

Mathematical modeling is now a standard tool for disease prevention and elimination efforts [10, 11]. There are very few mathematical models specific for MPX; yet, in recent years, even before the 2022 outbreak, the modelling activity has been picking up. In [12], the authors developed the first model to represent MPX and other pox-like infections. The model was later extended to include the coexistence of HIV and MPX [13]. Culling as a means to prevent MPX was investigated in [14]. In [15] and [16], the authors studied transmission dynamics with treatment and vaccination; in [17], the authors developed a model for diagnosing MPX and in [18], the authors performed stability analysis for equilibria of their ODE system. Other models include [19] where the authors studied quarantining and public education, [20] and [21] which concerns human-to-human transmissions. The global and local asymptotic stability and transmission dynamics were explored in [22]. The impact of smallpox vaccines on MPX epidemics was investigated in [23]. The fractional order ODE models were developed in [24] and [25]. A model incorporating sexual behavior dynamics and stratifying the population into high- and low-risk groups was developed in [26]. Stochastic models and individual based simulations for the current outbreak are also being developed [27–30].

The potential for the disease spread in the population can be measured by the basic reproduction number, *R*_0_, the number of secondary infections from a single infected individual in a susceptible population [31]. If interventions in disease transmission are implemented, the number of secondary infections from a single infected individual in an otherwise healthy population is called the effective reproduction number, *R*_*e*_ [32]. When *R*_0_, or more generally *R*_*e*_ is less than 1, then the disease cannot spread in the population [33].

### 1.3 Game theory

In the context of this paper, a game is a mathematical model of a situation where several individuals interact (directly or indirectly) with one another and where each individual acts in its own interest [34]. The game theory has a rich history; the modern treatments build on the ideas of John von Neumann [35] and John Nash [36]; see for example [37–40] or, for more biologically oriented applications, [41–45].

One way to define the solution of a non-cooperative game is the so-called Nash equilibrium (NE). In the NE, each player is assumed to know the equilibrium strategies of the other players, and no one has any incentive to change only their own strategy [36]. In this paper, we will focus on this concept of NE, but we note that there are other possible approaches, involving for example bounded rationality [46, 47] or quantal response equilibria [48, 49].

Game theoretical models proved themselves useful in studying complex epidemiological scenarios in which self-interested individuals take actions based on the decisions of the rest of the population [50, 51]; and, as argued in [52], by incorporating human behavior into the epidemiological models one can get better insight and predictions.

### 1.4 Vaccination games

Vaccination games are games in which individuals decide whether to vaccinate or not. They are a class of public goods games [53] because vaccination produces public goods (herd immunity against a disease) that have the following two main characteristics: non-rivalry, i.e., consumption of a good by one person does not affect other individuals, and non-exclusion of consumption, i.e., it is impossible to restrict the benefits to certain individuals [54]. Vaccination is prone to free-riding; the “free-riders” avoid the costs associated with vaccination while benefiting from vaccines taken by others [55]. People balance the perceived costs against the vaccine’s effectiveness [56] and it is well known that individuals act in a way that maximizes their self-interests, rather than the interests of the entire group [57].

The vaccination games have been applied to protection strategies to control diseases such as smallpox [58, 59], chickenpox [60], polio [61], influenza [62], Ebola [63], COVID-19 [64–66], chikungunya [67], Hepatitis B [68], lymphatic filiarisis [69]. [70] used the game theoretic framework to assess vaccination strategies with the presence of animal reservoirs of infection.

### 1.5 Content of the current paper

In the current paper, we extend the analysis from [70] by explicitly considering the MPX vaccine to be imperfect and allowing for the possibility of infections after vaccination. We focus on human-to-human transmission as done in [21]. We study Nash equilibria, the solutions of the vaccination game in which susceptible individuals decide whether or not to vaccinate against MPX. We calibrate our model based on historical data about MPX and we also use data from the 2022 outbreaks. The analysis reveals a possibility of multiple Nash equilibria and the existence of backward bifurcation. We perform sensitivity analysis and also study a hypothetical scenario under which the MPX transmission rate is higher than generally assumed based on the historical data. We demonstrate that the voluntary vaccination alone will not be enough to substantially limit the spread of MPX.

## 2 Model and methods

We adapt a SVEIR compartmental model of MPX [21] and extend it by incorporating the game-theoretic framework of voluntary vaccination as done in [71].

Individuals are born susceptible (*S*) at rate Λ. Without vaccination, a susceptible individual becomes exposed (*E*) after coming in contact with an infectious individual (*I*); this happens at rate $\beta \frac{I}{N}$ where *N* is the population size. The incubation period lasts *σ*^−1^ after which the individual develops MPX. The MPX cases recover (*R*) at rate *γ*^−1^ and gain permanent immunity.

The original model presented in [21] allowed for a proportion of individuals to be vaccinated at birth. However, since the smallpox vaccine is no longer mandated at birth, it is more realistic to consider that the vaccination occurs later in life. As in [65] for the COVID-19 vaccine, we will assume the susceptible individuals are vaccinated (*V*) at rate *ψ*. While in theory, *ψ* ∈ [0, ∞), there are bounds on how fast the population can be vaccinated. We will thus assume *ψ* ∈ [0, *ψ*_max_] where *ψ*_max_ is the maximal feasible vaccination rate.

The vaccine does not provide complete protection; the vaccine efficacy is *e* ∈ (0, 1]. It follows that the vaccinated individuals become exposed at rate $\left(1-e\right)\beta \frac{I}{N}$.

Finally, all individuals can die of natural causes at a rate *μ*.

**Fig 1:**
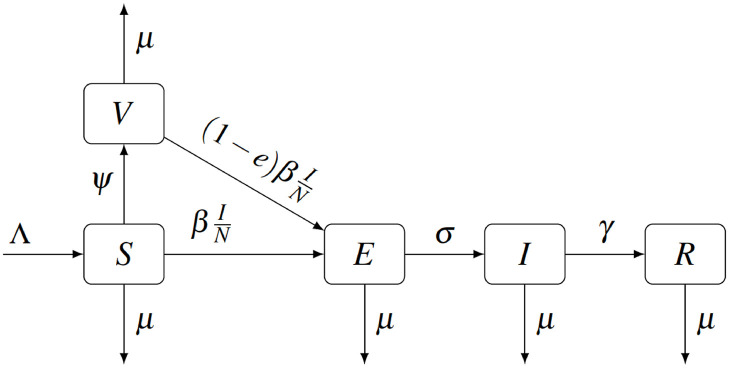
Scheme of the SVEIR model for MPX.

This compartmental model is illustrated in Fig 1. We note that this ODE model is a special case of a SARS model considered in [74]. The notation is explained and the parameter values are shown in Table 1. The model calibration is explained in detail in Section 4.

**Table 1:** Model parameters. Times are in days, rates are per capita per day (and Λ is individuals per day). Details on model calibration are shown in Section 4.

Notation	Meaning	Base value	Range	Reference(s)
*μ*	Natural death rate	$\frac{0.01027}{365}$	$\left[\frac{0.005}{365},\frac{0.02}{365}\right]$	[72]
Λ	Natural birth rate	$\frac{0.011}{365}$	$\left[\frac{0.005}{365},\frac{0.02}{365}\right]$	[73]
*β*	Transmission rate	0.09	[0.045, 0.18]	[21]
*γ* ^−1^	Infectious period	23.5	[15, 32]	[2]
*e*	Vaccine efficacy	0.85	[0.82, 0.9]	[4]
*σ* ^−1^	Incubation period	12	[7, 17]	[2]
*ψ*	Vaccination rate	variable	[0, *ψ*_max_]	
*ψ*_max_	Maximal feasible vaccination rate	$\frac{1}{365}$	N/A	Assumed
*C*_*V*_	Cost of vaccination	1	N/A	Set as a unit
*C*_*MPX*_	Cost of MPX infection	2.5	[1, 10]	Assumed
$C_{MPX}^{*}$	Relative cost of MPX	$\frac{C_{MPX}}{C_{V}}$		

We extend this ODE model of MPX transmission by incorporating the game-theoretic component as done in [71] for measles, smallpox, and other childhood diseases or in [65] for the recent COVID-19 outbreak or [63] for an Ebola outbreak.

A vaccination game is played by susceptible individuals who are assumed to be rational, acting in their own best interests, and having complete information about the MPX epidemic. The individuals decide whether to vaccinate or stay unvaccinated. The payoff to the individual is a function that depends on the action of that individual (whether they vaccinate or not) and the actions of other players (how fast they are vaccinating as a whole). The payoff incorporates the cost of the vaccination, *C*_*V*_, the risk of getting infected, *π*_*NV*_ and *π*_*V*_ evaluated below, and the costs of the MPX infection $C_{MPX}^{*}$.

To evaluate the probability of getting exposed to MPX, we follow [71]. We assume that the epidemics reached a steady state with I* infected individuals. The formula for I* is given later by (15); it is important to note that I* depends on *ψ*, the vaccination rate in the population, but not on the decision of the focal individual.

The probability that an unvaccinated individual becomes exposed to MPX is

$$\begin{array}{c} \pi_{NV}=\frac{\beta \frac{I^{*}}{N}}{\beta \frac{I^{*}}{N}+\mu }, \end{array}$$ (1)

where $\beta \frac{I^{*}}{N}+\mu$ is a rate at which individuals with no intention to vaccinate leave the Susceptible compartment; $\beta \frac{I^{*}}{N}$ is the rate at which they enter the Exposed compartment.

Similarly, the probability that a vaccinated individual becomes exposed to MPX is

$$\begin{array}{c} \pi_{V}=\frac{\left(1-e\right)\beta \frac{I^{*}}{N}}{\left(1-e\right)\beta \frac{I^{*}}{N}+\mu }. \end{array}$$ (2)

Once exposed, the individual will become infected with probability $\frac{\sigma }{\sigma +\mu }$.

The solution of the vaccination game, the Nash equilibrium (NE), is the population vaccination rate *ψ*_*NE*_ such that in this situation no individual has an incentive to deviate from the population strategy, i.e. either (1) *ψ*_*NE*_ = 0 when *π*_*NV*_ < *π*_*V*_ for *ψ* = 0, (2) *ψ*_*NE*_ = *ψ* if *π*_*NV*_ = *π*_*V*_ when the vaccination rate is *ψ*, or (3) *ψ*_*NE*_ = *ψ*_max_ if *π*_*NV*_ > *π*_*V*_ when the vaccination rate is *ψ* = *ψ*_max_.

## 3 Analysis

### 3.1 Analysis of the ODE system

The model yields the following differential equations:

$$\begin{array}{c} \frac{dS}{dt}=\Lambda -(\psi +\mu +\beta \frac{I}{N})S \end{array}$$ (3)

$$\begin{array}{c} \frac{dV}{dt}=\psi S-(\mu +\left(1-e\right)\beta \frac{I}{N})V \end{array}$$ (4)

$$\begin{array}{c} \frac{dE}{dt}=(\frac{\beta }{N}S+\left(1-e\right)\frac{\beta }{N}V)I-\left(\mu +\sigma \right)E \end{array}$$ (5)

$$\begin{array}{c} \frac{dI}{dt}=\sigma E-(\mu +\gamma )I \end{array}$$ (6)

$$\begin{array}{c} \frac{dR}{dt}=\gamma I-\mu R. \end{array}$$ (7)

There are two equilibria of the dynamics (3)–(7). The disease-free equilibrium (DFE) $E^{0}=\left(S^{0},V^{0},E^{0},I^{0},R^{0}\right)$ is given by $\left(\frac{\Lambda }{\mu +\psi },\frac{\Lambda \psi }{\mu (\mu +\psi )},0,0,0\right)$, i.e., $S^{0}=N\frac{\mu }{\mu +\psi }$ and $V^{0}=N\frac{\psi }{\mu +\psi }$ where $N=\frac{\Lambda }{\mu }$ is the total population size. [74] derived the effective reproduction number, i.e., the number of secondary infections from a single infected individual in an otherwise healthy population, as

$$\begin{array}{c} R\left(\psi \right)=R_{0}\frac{\mu +(1-e)\psi }{\psi +\mu }. \end{array}$$ (8)

Here,

$$\begin{array}{c} R_{0}=\frac{\sigma \beta }{\left(\sigma +\mu )(\gamma +\mu \right)} \end{array}$$ (9)

is the basic reproduction number, i.e., the number of secondary infections from a single infected individual in an otherwise healthy and *unvaccinated* population.

The DFE is globally asymptotically stable if *R*(*ψ*) ≤ 1 [74, Theorem 4.1].

Let *ψ*_*HI*_ be the minimal level of vaccination needed for achieving a herd immunity; specifically let *ψ*_*HI*_ ∈ [0, ∞) be such that *R*(*ψ*)≤1 for all *ψ* ≥ *ψ*_*HI*_. It follows that DFE is the only stable equilibrium for *ψ* ≥ *ψ*_*HI*_ and

$$\begin{array}{c} \psi_{HI}=\{\begin{array}{cc} 0 & \;\text{if}\;R_{0}\leq 1,\\ \infty & \;\text{if}\;e\leq 1-\frac{1}{R_{0}},\\ \frac{R_{0}-1}{1-\left(1-e\right)R_{0}}\mu & \;\text{otherwise.} \end{array} \end{array}$$ (10)

In particular, if $e\leq 1-\frac{1}{R_{0}}$, then no vaccination rate will prevent the epidemic. Moreover, if *ψ*_*HI*_ > *ψ*_max_, then no feasible vaccination rate can prevent the epidemic.

The endemic equilibrium exists only when *R*(*ψ*) > 1. Moreover, the endemic equilibrium is locally asymptotically stable whenever *R*(*ψ*) > 1. The theoretical results and simulations performed in [74] suggest that it is globally asymptotically stable when *R*(*ψ*) > 1 and the initial population satisfies *E*|_*t* = 0_ > 0 or *I*|_*t* = 0_ > 0. These results are also supported by [75] who studied a similar model without the *E* compartment.

Thus, for any set of parameter values, there is only one stable equilibrium of the ODE system. Let us denote it by $E^{*}=\left(S^{*},E^{*},I^{*},R^{*},V^{*}\right)$. As done in [74], setting the derivatives of (3)–(7) to 0 and solving the resulting system of algebraic equation yields a polynomial equation for I* in the form

$$\begin{array}{c} I^{*}\left(aI^{*2}+bI^{*}+c\right)=0 \end{array}$$ (11)

where

$$\begin{array}{c} a={\left(\frac{\beta }{N}\right)}^{2}\left(1-e\right) \end{array}$$ (12)

$$\begin{array}{c} b=\frac{\beta }{N}\mu (1+\frac{\left(1-e)(\psi +\mu \right)}{\mu +(1-e)\psi }(1-R\left(\psi \right)+\left(1-e\right)\frac{\psi }{\mu })) \end{array}$$ (13)

$$\begin{array}{c} c=\mu (\psi +\mu )(1-R(\psi )). \end{array}$$ (14)

The equation aI*^2^ + bI* + *c* = 0 has no positive root when *R*(*ψ*) < 1. Thus, if *R*(*ψ*) ≤ 1, I* = 0 is the only biologically relevant solution of (11). On the other hand, as seen above, when *R*(*ψ*) > 1, the disease-free equilibrium corresponding to I* = 0 is not stable. Thus, we have

$$\begin{array}{c} I^{*}=\{\begin{array}{cc} 0, & \text{if}\;R(\psi )\leq 1\text{,}\;\text{i.e.,}\;\text{if}\;\psi \geq \psi_{HI},\\ -\frac{c}{b}, & \text{if}\;R(\psi )>1\text{,}\;\text{and}\;e=1,\\ \frac{-b+\sqrt{b^{2}-4ac}}{2a}, & \text{otherwise}, \end{array} \end{array}$$ (15)

Note that I* is always non-increasing in *ψ* and it is decreasing in *ψ* whenever I* > 0 (and *e* > 0).

Furthermore, it follows easily from the algebra that

$$\begin{array}{c} S^{*}=\frac{\Lambda }{\beta \frac{I^{*}}{N}+\psi +\mu } \end{array}$$ (16)

$$\begin{array}{c} E^{*}=\frac{\mu +\gamma }{\sigma }I^{*} \end{array}$$ (17)

$$\begin{array}{c} R^{*}=\frac{\gamma }{\mu }I^{*} \end{array}$$ (18)

$$\begin{array}{c} V^{*}=\frac{\psi S^{*}}{\left(1-e\right)\beta \frac{I^{*}}{N}+\mu }. \end{array}$$ (19)

### 3.2 Solving the vaccination game

Let $C_{MPX}^{*}=\frac{C_{MPX}}{C_{V}}$ be the expected cost of MPX infection expressed relative to the cost of the vaccine. The incentive function *h*(*ψ*) for an individual to vaccinate when the population vaccination rate is *ψ* is given by a difference between the expected costs if not vaccinated and the expected costs if vaccinated (including the potential cost of contracting MPX), i.e., we can assume

$$\begin{array}{c} h\left(\psi \right)=C_{MPX}^{*}(\frac{\sigma }{\sigma +\mu })\left(\pi_{NV}-\pi_{V}\right)-1. \end{array}$$ (20)

Here, $\left(\frac{\sigma }{\sigma +\mu }\right)$ is the probability an exposed individual becomes infected. It follows that the Nash equilibrium is given by

$$\begin{array}{c} \psi_{NE}=\{\begin{array}{cc} 0, & \;\text{if}\;h(0)<0,\\ \text{root(s)}\;\text{of}\;h, & \;\text{if}\;\text{in}\;[0,\psi_{\text{max}}],\\ \psi_{\text{max}}, & \;\text{if}\;h(\psi_{\text{max}})>0. \end{array} \end{array}$$ (21)

### 3.3 Calculations of the Nash equilibria

Here we show detailed calculations for the Nash equilibria of the vaccination game and find the roots of *h*(*ψ*) = 0. We will study the function

$$\begin{array}{c} \tilde{h}\left(x\right)=(\frac{C_{MPX}^{*}\sigma }{\sigma +\mu })(\frac{x}{x+1}-\frac{(1-e)x}{(1-e)x+1})-1 \end{array}$$ (22)

$$\begin{array}{cc} & =\frac{(\frac{C_{MPX}^{*}\sigma }{\sigma +\mu })\;xe}{\left(x+1)((1-e)x+1\right)}-1. \end{array}$$ (23)

Note that $h\left(\psi \right)=\tilde{h}(\frac{\beta }{\Lambda }I^{*})$; and while we are primarily interested in the behavior of *h*(*ψ*) on [0, *ψ*_*HI*_), we will investigate the function $\tilde{h}\left(x\right)$ on [0, ∞).

It follows that $\tilde{h}\left(x\right)⋚0$ if and only if

$$\begin{array}{c} 0⋚\left(1-e\right)x^{2}+[2-e(1+\frac{C_{MPX}^{*}\sigma }{\sigma +\mu })]\;x+1. \end{array}$$ (24)

We will assume $\frac{C_{MPX}^{*}\sigma }{\sigma +\mu }>1$, as otherwise the right-hand side of (24) is positive for *x* ≥ 0. Note that this is a reasonable assumption; *μ* ≪ *σ* and thus $\frac{\sigma +\mu }{\sigma }\approx 1$ and if $C_{MPX}^{*}<1$, then the vaccine would cost more than the disease, i.e., nobody would vaccinate. We will also assume ${\left(2-e\left(1+\frac{C_{MPX}^{*}\sigma }{\sigma +\mu }\right)\right)}^{2}-4\left(1-e\right)>0$, i.e.,

$$\begin{array}{c} \frac{4\frac{C_{MPX}^{*}\sigma }{\sigma +\mu }}{{\left(1+\frac{C_{MPX}^{*}\sigma }{\sigma +\mu }\right)}^{2}}\leq e<1. \end{array}$$ (25)

When (25) does not hold, there are no real roots of $\tilde{h}$ and the right-hand side of (24) is positive for *x* ≥ 0. When (25) holds, the roots of $\tilde{h}$ are given by

$$\begin{array}{c} x_{1,2}=\frac{e\;(1+\frac{C_{MPX}^{*}\sigma }{\sigma +\mu })-2\pm \sqrt{{\left(e(1+\frac{C_{MPX}^{*}\sigma }{\sigma +\mu })-2\right)}^{2}-4\left(1-e\right)}}{2(1-e)}. \end{array}$$ (26)

Setting (15) equal to $I_{1,2}^{*}=\frac{\Lambda }{\beta }x_{1,2}$ where *x*_1,2_ is given by (26), we obtain

$$\begin{array}{c} -\mu^{2}\left(1-e\right)x_{1,2}^{2}=\mu \left(\psi +\mu \right)\left(1-R\left(\psi \right)\right)+\mu^{2}(1+\frac{\left(1-e)(\psi +\mu \right)}{\mu +(1-e)\psi }(1-R\left(\psi \right)+\left(1-e\right)\frac{\psi }{\mu }))\;x_{1,2}. \end{array}$$ (27)

Simplifying (27) and solving for *ψ* yields

$$\begin{array}{c} \psi_{1,2}=\frac{\mu (-\left(1-e\right)x_{1,2}^{2}+\left(\left(1-e\right)\left(R_{0}-1\right)-1\right)x_{1,2}+R_{0}-1)}{1+\left(1-e\right)\left(x_{1,2}-R_{0}\right)}. \end{array}$$ (28)

### 3.4 Analysis of the Nash equilibria

In the context of vaccination games studied in this paper, there are two kinds of NE. If *h*(*ψ*_*NE*_) = 0 and *h*′(*ψ*_*NE*_) < 0, then *ψ*_*NE*_ is called a convergent stable Nash equilibrium (CSNE) [71]. If the population adopts a strategy *ψ* ≈ *ψ*_*NE*_, then it will evolve even closer to *ψ*_*NE*_. For similar reasons, if *h*(0) < 0 or if *h*(*ψ*_max_) > 0, then 0 or *ψ*_max_ are CSNEs. However, when *h*(*ψ*_*NE*_) = 0 and *h*′(*ψ*_*NE*_) > 0, then *ψ*_*NE*_ is not CSNE, as a small deviation from *ψ*_*NE*_ will result in even larger deviation.

We note that there is a difference between the usual vaccination games (such as [70, 71]) that assume *e* = 1 and our more general case with *e* < 1. If *e* = 1, the incentive function *h*(*ψ*) is decreasing in *ψ* and *ψ*_*HI*_ < ∞. Thus, assuming *ψ*_*HI*_ < *ψ*_max_, there is *ψ* > *ψ*_*HI*_ for which I* = 0 and *h*(*ψ*) = −1 < 0. Consequently, there is at most one root of *h*(*ψ*) which exists if and only if *h*(0) ≥ 0. However, when *e* < 1, there can be multiple roots of *h* and also multiple Nash equilibria as demonstrated later.

### 3.5 Uncertainty and sensitivity analysis

We performed uncertainty and sensitivity analysis using the Latin hyper-cube sampling with partial rank correlation coefficient (LHS-PRCC) scheme [76, 77]. The scheme is described in detail in [78] and their MATLAB and R implementation can be found in [79]. Our MATLAB code, including the code for uncertainty and sensitivity analysis, is in the S1 Code.

The Latin Hyper-cube Sampling (LHS) is a stratified sampling without replacement; the random parameter distributions are divided into intervals of equal probability and the sampling is independent for each parameter. This method provides an unbiased estimate of the average model output while it requires fewer samples than simple random sampling to achieve the same accuracy [80].

The partial rank correlation coefficient (PRCC) between model parameter *p* and model output *O* is a correlation coefficient

$$\begin{array}{c} r_{R_{p},R_{O}}=\frac{\text{Cov}(R_{p},R_{O})}{\sqrt{\text{Var}\left(R_{p}\right)\text{Var}\left(R_{O}\right)}} \end{array}$$ (29)

between *R*_*p*_ and *R*_*O*_ which are residuals of the rank-transformed linear regression models for *p* and *O*. PRCC is a robust sensitivity measure for nonlinear but monotonic relationships between inputs and the output, as long as little to no correlation exists between the inputs [78].

## 4 MPX calibration

### 4.1 Demographic parameters

We used data from the CDC to establish the natural birth rate and natural death rate. From [73], the birth rate in the U.S. in 2020 was 11.0 per 1,000. We will thus assume $\mu =\frac{11}{1000\cdot 365}$ per individual per day. From [72], the death rate in the U.S. in 2020 was 1,027.0 deaths per 100,000 population. We will thus assume $\Lambda =\frac{0.01027}{365}$.

### 4.2 Disease progression

The MPX incubation period lasts 7–17 days [2]. We thus assume *σ*^−1^ = 12 days on average.

The prodromal period lasts 1–4 days [2] and the rash period lasts 14–28 days [2]. We thus assume that *γ*^−1^ = 23.5 days on average.

### 4.3 MPX related mortality

For the simplicity of the ODE model and its analysis, we do not consider any MPX related mortality. While the mortality was reported to be as high as 11% [4], and in recent times the case fatality has been 3–6% [81], the current 2022 outbreak has over 6000 reported cases of MPX and only 3 deaths [6]. We note that [82] reports 66 deaths in African countries and there may be a time lag in death reporting. Thus, there may be a non-negligible mortality even in the current outbreak. Yet, the mortality is likely relatively small to make substantial impacts on the main conclusions.

### 4.4 Vaccine efficacy

Data from Equateur Province of Democratic Republic of Congo from 1981 to 1986, in the years following smallpox eradication, suggest that smallpox vaccine conferred 85% protection against monkeypox [2]. For the purpose of this paper, we will thus assume *e* ≈ 0.85.

The data are described in detail in [4]. Within the household contacts, the attack rate among unvaccinated individuals was 9.28 and among the vaccinated individuals it was 1.31 [4, Table 5]. Thus, the vaccine efficiency was estimated as $1-\frac{1.31}{9.28}\approx 0.855$. However, the numbers within the house differ by gender. For males, the efficacy was $1-\frac{0.85}{8.61}\approx 0.9$ while for females the efficacy was $1-\frac{1.74}{9.91}\approx 0.82$.

Given that in the 2022 MPX outbreak, 99.5% (4385/4406) of cases for which the sex is known are men [6], it may be tempting to adopt *e* = 0.9 based on the above calculations. However, the nature of contacts for data collected in [4] was likely different from the nature of contact in the 2022 outbreak in MSM.

The 3rd generation smallpox vaccine Imvanex (Modified Vaccine Ankara—MVA) has been authorised by the European Medicines Agency, but scientific evidence on the vaccine effectiveness of MVA against MPX is still lacking [84]. Overall, a more recent estimate of vaccine efficacy is needed.

### 4.5 Basic reproduction number

While the basic reproduction number is not a parameter of the model, we will use the estimate of *R*_0_ to derive an estimate of *β* by *R*_0_*γ* as in a section outlined below.

Using data collected in the Democratic Republic of the Congo between 1966–1984, [21] estimated the basic reproduction number for monkeypox as *R*_0_ ≈ 2.13 with bounds between 1.46 and 2.67.

To estimate *R*_0_ for the 2022 MPX outbreak, we used data from [85], who shared their raw data set of MPX cases on github [86]. We used SAS to obtain incidence rates for the whole world as well as for Spain, England, and Germany, which were the three countries with the most cases; the SAS code is provided in the S2 Code.

We used an online tool [83] to estimate *R*_0_ from the incidence data as follows. We set the sliding window to 7 days, set prior mean value for *R*_0_ to 2.1, and prior SD to 0.3. We assumed all transmissions to be local (for the lack of better data). We used the serial interval by distributional estimate, using the option “Parametric without uncertainty” and set the mean to 9.7, and SD to 0.5 [87]. The results are summarized in Table 2. While the estimates vary and generally decrease in time, there are some relatively high values of *R*. This leaves the possibility of *R*_0_ > 3 open at least for the current 2022 outbreak.

**Table 2:** Estimated effective reproduction number for the 2022 MPX outbreak using method from [83] on MPX reported cases data from [85].

	World		Spain		England		Germany	
Day	Mean	SD	Mean	SD	Mean	SD	Mean	SD
10	6.27	0.50	2.07	0.18	2.67	0.33	2.93	0.29
15	11.37	0.61	1.47	0.13	4.80	0.40	3.06	0.27
20	3.54	0.17	1.33	0.11	4.11	0.32	2.02	0.16
25	1.71	0.07	2.66	0.16	1.48	0.12	2.32	0.15
30	1.38	0.06	2.20	0.14	1.35	0.10	2.75	0.15
35	1.54	0.05	1.19	0.07	1.81	0.11	2.00	0.10
40	2.22	0.06	2.17	0.10	2.26	0.12	1.56	0.07
45	1.81	0.05	1.83	0.08	1.67	0.09	1.25	0.06
50	1.76	0.04			1.56	0.07		
55	1.36	0.03			0.74	0.04		

### 4.6 Transmission rate

By (9), and assuming *μ* ≪ *σ* and *μ* ≪ *γ*,

$$\begin{array}{c} R_{0}=\frac{\sigma \beta }{\left(\sigma +\mu )(\gamma +\mu \right)}\approx \frac{\beta }{\gamma }. \end{array}$$ (30)

Thus,

$$\begin{array}{c} \beta \approx R_{0}\gamma . \end{array}$$ (31)

Based on estimates of *R*_0_ from [21] discussed above, we get *β* ≈ 2.13/23.5 ≈ 0.09 with bounds between 1.47/32 ≈ 0.045 and 2.67/14 ≈ 0.18. We note that this estimate agrees with a relatively crude estimate from [70] who used transmission risk data from [88] to arrive at the same value.

### 4.7 Validation

To validate our choice of parameters and their ranges, we run the sensitivity analysis of the basic reproduction number on the parameter values and ranges as specified above and in Table 1.

**Fig 2:**
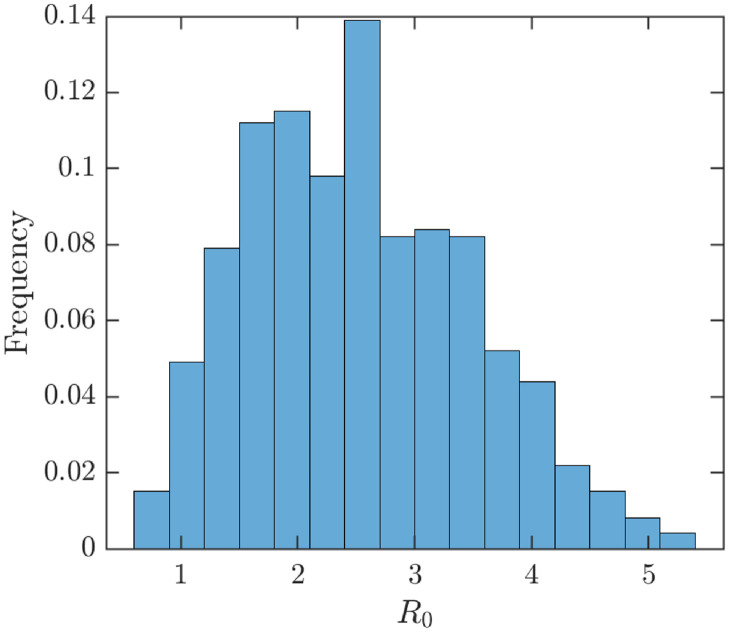
Uncertainty analysis of *R*_0_. Parameter values and ranges as specified in Table 1. The average value of *R*_0_ is about 2.53.

**Fig 3:**
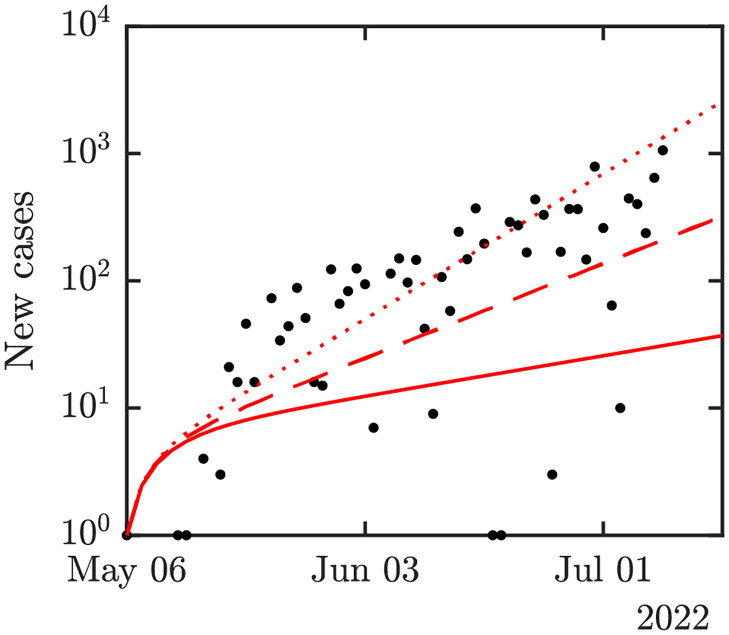
Actual (black dots) and predicted incidence. Full red line is for *β* = 0.09, dashed is for *β* = 0.19 and dotted is for *β* = 0.29. All other parameters are as specified in Table 1.

The estimated basic reproduction number is *R*_0_ ≈ 2.11, and the average value during the uncertainty analysis was 2.53; see Fig 2. This value seemed reasonable and in agreement with historical data; see Fig 3.

We also plotted the actual new MPX cases as obtained from [85] against the model’s predicted incidence. We numerically solved the system (3)–(7). The number of new cases at day *d* was obtained as *σE*|_*t*=*d*_. We normalized it so that at the start of the epidemic we have *σE*|_*t*=0_ = 1; see Fig 3. We note that the best match appears for *β* ≈ 0.29. However, this would yield *R*_0_ ≈ 6.8 which is more in the order of *R*_0_ for smallpox [89]. The fit is still good enough for *β* ≈ 0.18 which yields *R*_0_ ≈ 4.23. This is still larger than the usual estimates for MPX. However, as shown in Table 2, such a value of *R*_0_ is not completely unreasonable for the 2022 MPX outbreak. Consequently, while we will do most of our calculations for *β* = 0.09 which seems to be in agreement with all previous estimates and historical data, we will also consider *β* = 0.18, the upper bound for *β* estimates.

### 4.8 Maximal feasible vaccination rate

We did not locate any data on the maximal feasible vaccination rate. We will assume that the population can get vaccinated about once a year, i.e., *ψ*_max_ = 1/365. In the U.S., at the time of writing, the demand for the vaccine exceeds the supply [90] and for large populations, the vaccine supply is likely the most important factor limiting the vaccination rate. However, even for smaller populations and/or during the times the vaccine supply will be restored, there may be logistical issues (such as limited supply of qualified nurses and doctors) preventing a significantly faster vaccination rate.

### 4.9 Costs of vaccination and costs of MPX

There can be many types of costs associated with vaccination, including the actual cost for vaccination, time loss, and travel cost that all have negative effects on the probability of complete vaccination [91].

Similarly, the expected cost of the disease *C*_*MPX*_ includes possible medication costs, doctor charges, time loss and similar direct and indirect costs.

We were not able to locate any reliable and accurate values for *C*_*V*_ and *C*_*MPX*_ applicable to the current outbreak. We note that [70] used *C*_*V*_ = 4 based on [92] and *C*_*MPX*_ = 100 based on [93]. However, those values are for the Democratic Republic of the Congo and based on historical data. In the current 2022 outbreak, especially in the U.S., the vaccine is provided for free, and the cost is thus limited to indirect costs such as taking the time off work to get vaccinated and possibly dealing with minor vaccine side effects [94]. At the same time, the MPX infection does not seem to add any extra monetary expenses to the individuals, apart from mild symptoms and taking the time off from work although cases of severe pain have been reported. We will thus assume $C_{MPX}^{*}$ to be somewhere between 1 and 10 and note that in reality, the value will likely be different from person to person.

The value of $C_{MPX}^{*}$ plays a role in the incentive function given in (20). The formula contains $\left(\frac{\sigma }{\sigma +\mu }\right)$, the probability an exposed individual becomes infected, which, for MPX parameters discussed above, is approximately 1. Here, we can assume that $C_{MPX}^{*}$ is not affected by natural mortality. However, if the MPX infection lasts significantly longer, one would have to carefully account for the effects of natural mortality.

Furthermore, we note that while the real costs are important, what truly matters for the individuals are the perceived costs of the disease and vaccination; yet the actual model does not change when we change the interpretation of $C_{MPX}^{*}$ from “real” to “perceived” relative cost.

## 5 Results

**Fig 4:**
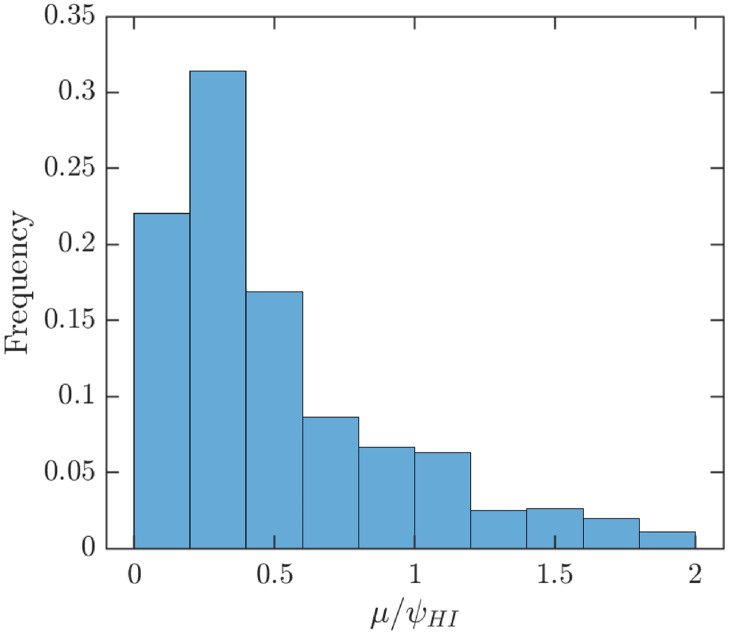
Uncertainty analysis of *μ*/*ψ*_*HI*_. Parameter values and ranges as specified in Table 1. For those values, *μ*/*ψ*_*HI*_ ≈ 0.61 meaning that to achieve herd immunity, the whole population at risk has to be vaccinated in about 61% of the average lifespan. The average value of *μ*/*ψ*_*HI*_ is about 0.53.

**Fig 5:**
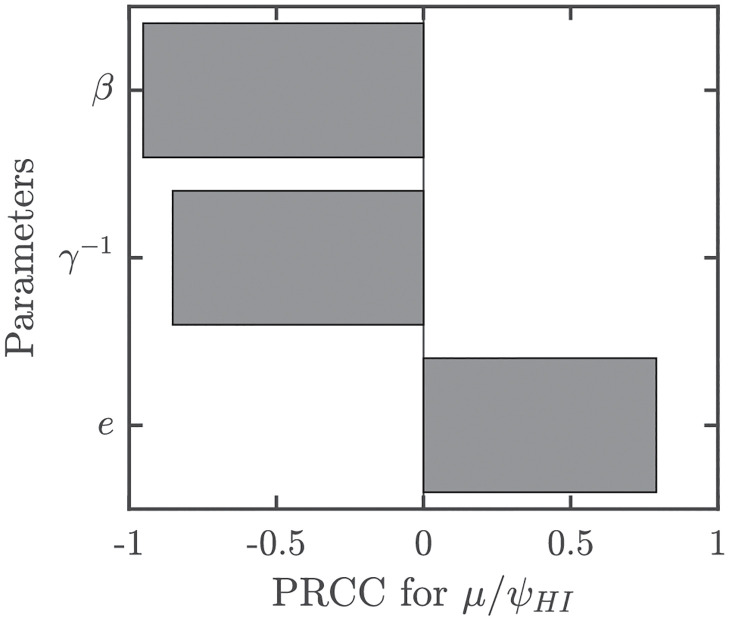
Sensitivity analysis of *μ*/*ψ*_*HI*_. Parameter values and ranges as specified in Table 1. For those values, *μ*/*ψ*_*HI*_ ≈ 0.61 meaning that to achieve herd immunity, the whole population at risk has to be vaccinated in about 61% of the average lifespan.

Historical data indicate that MPX outbreaks can be eliminated by vaccination. Indeed, the estimates for the vaccine efficacy *e* ≈ 0.85 and basic reproduction number *R*_0_ ≈ 2.13 based on data from 1966–1984 [21] mean that $e>1-\frac{1}{R_{0}}$. Thus, the vaccination rate needed to achieve herd immunity, *ψ*_*HI*_, is finite. For the parameter values considered in Table 1, we get *μ*/*ψ*_*HI*_ ≈ 0.61, meaning that if the whole population at risk can be vaccinated in about 61% of the average lifespan, then herd immunity will be reached. Fig 4 illustrates the uncertainty and Fig 5 shows the sensitivity analysis of *μ*/*ψ*_*HI*_. It shows a natural result that in order to achieve MPX elimination, the vaccination has to be done faster if the transmission rate, *β*, or the infectious period, *γ*^−1^, increase. On the other hand, the vaccination can be done slower if the vaccine efficacy, *e*, increases.

**Fig 6:**
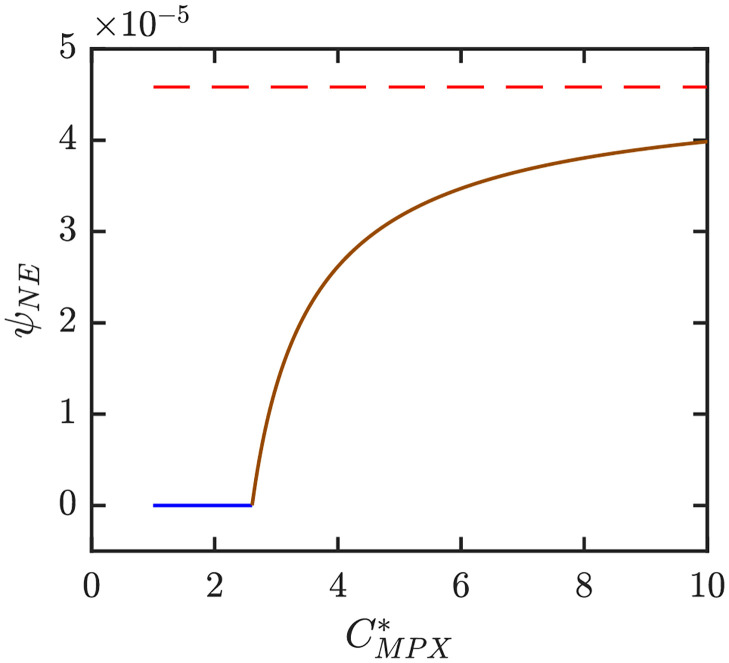
The optimal voluntary vaccination rates. The lines are color coded corresponding to the regions shown in Fig 9 which shows a diagram for Nash equilibria as *e* and $C_{MPX}^{*}$ vary. Blue: 0 is the only NE and it is CSNE. Brown: positive *ψ*_*NE*_ is the only NE and it is CSNE. Red dashed line shows the value of *ψ*_*HI*_. Unless varied or otherwise specified, the parameters are as in Table 1.

**Fig 7:**
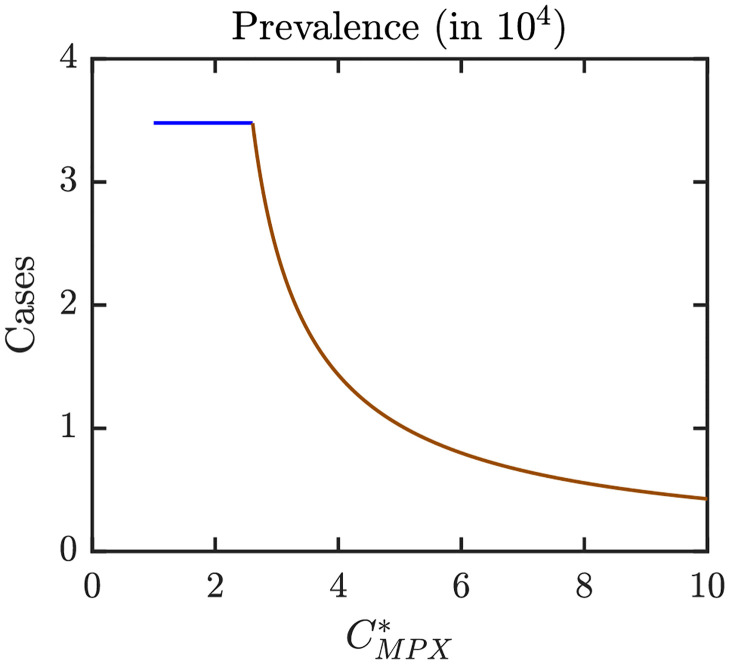
MPX prevalence in a population that uses optimal voluntary vaccination rates. The lines are color coded corresponding to the regions shown in Fig 9 which shows a diagram for Nash equilibria as *e* and $C_{MPX}^{*}$ vary. Blue: 0 is the only NE and it is CSNE. Brown: positive *ψ*_*NE*_ is the only NE and it is CSNE. Unless varied or otherwise specified, the parameters are as in Table 1.

**Fig 8:**
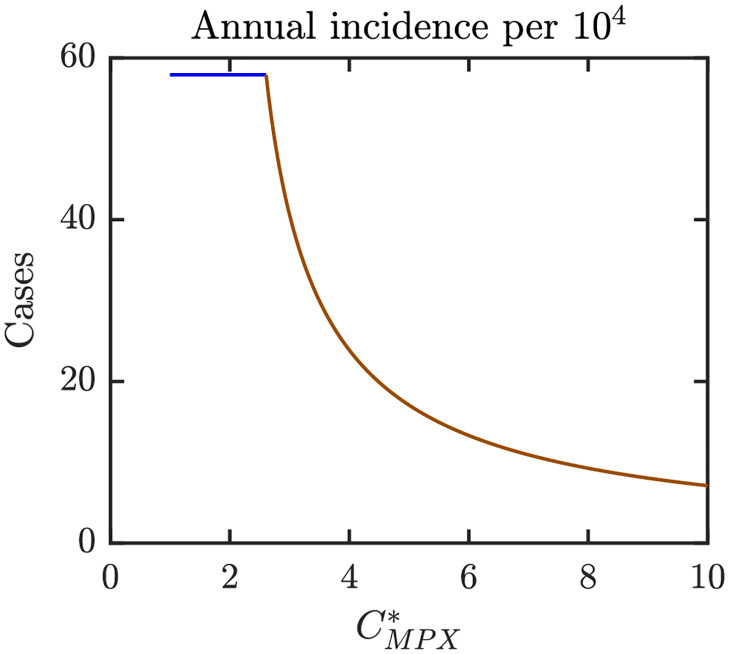
Annual MPX incidence in a population that uses optimal voluntary vaccination rates. The lines are color coded corresponding to the regions shown in Fig 9 which shows a diagram for Nash equilibria as *e* and $C_{MPX}^{*}$ vary. Blue: 0 is the only NE and it is CSNE. Brown: positive *ψ*_*NE*_ is the only NE and it is CSNE. Unless varied or otherwise specified, the parameters are as in Table 1.

Figs 6–8 illustrate the Nash equilibrium values, the MPX prevalence, and the annual MPX incidence in the population using the optimal voluntary vaccination rates. For the parameters as in Table 1, the NE is to “not vaccinate” as long as $C_{MPX}^{*}$, the cost of MPX relative to the cost of vaccine, is less than about 2.6. Without vaccination, the prevalence would be about 3.5 cases per 10^4^ individuals. However, even with optimal voluntary vaccination and relatively high $C_{MPX}^{*}\approx 10$, the MPX prevalence in the equilibrium is more than 0.5 cases per 10^4^ population. The annual incidence without any vaccination is almost 60 cases per 10^4^; and even with optimal voluntary vaccination and $C_{MPX}^{*}\approx 10$, the incidence would stay around 10 cases per 10^4^ population.

**Fig 9:**
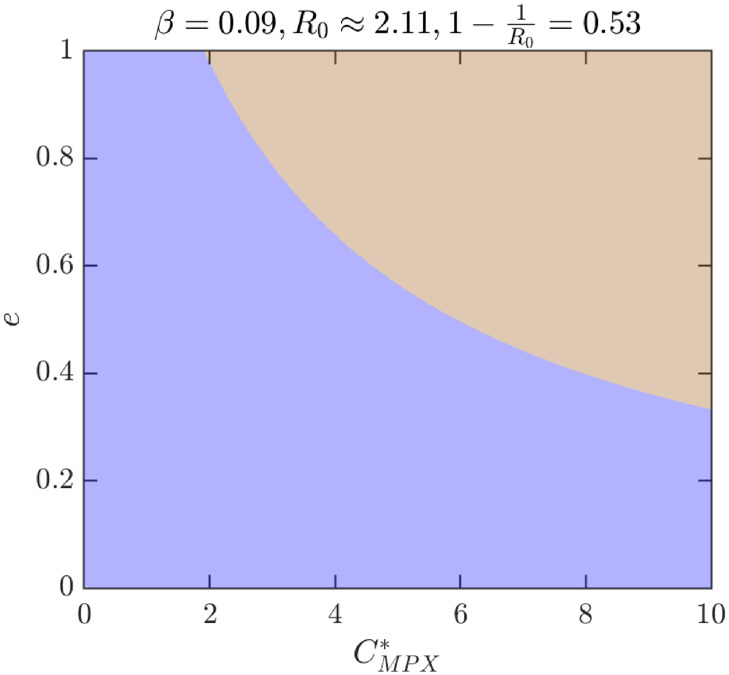
Nash equilibria as *e* and $C_{MPX}^{*}$ vary. Blue: 0 is the only NE and it is CSNE. Brown: positive *ψ*_*NE*_ is the only NE and it is CSNE. Unless varied or otherwise specified, the parameters are as in Table 1.

**Fig 10:**
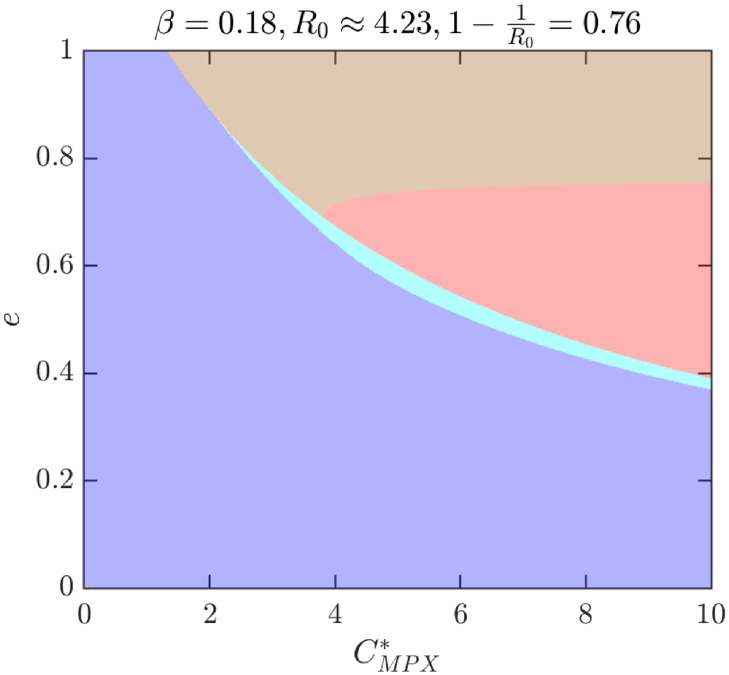
Nash equilibria as *e* and $C_{MPX}^{*}$ vary for higher transmission rate, *β* = 0.18. Other parameters are as in Table 1 unless they vary or are otherwise specified. Blue: 0 is the only NE and it is CSNE. Brown: positive *ψ*_*NE*_ < *ψ*_max_ is the only NE and it is CSNE. Light blue: three NEs, 0 and the larger NE are CSNE. Red: maximal feasible vaccination rate is the only CSNE.

**Fig 11:**
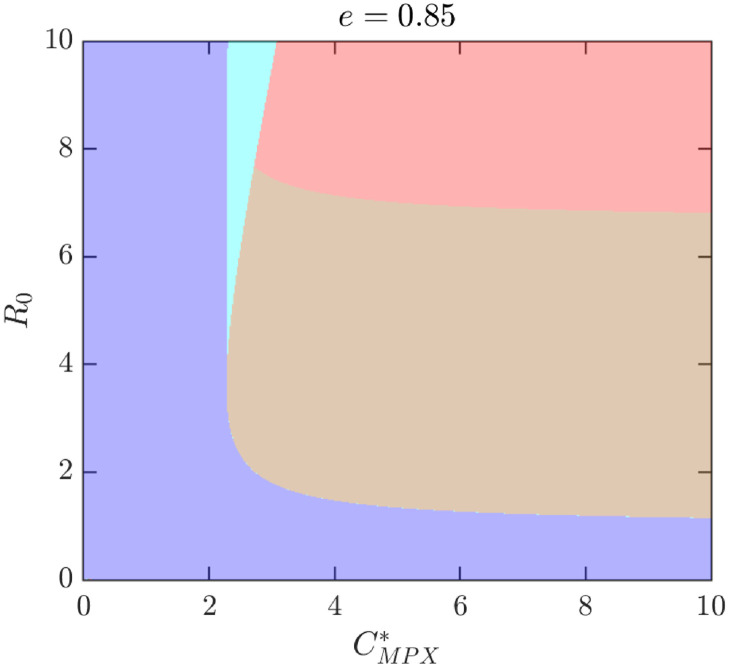
Nash equilibria as *R*_0_ and $C_{MPX}^{*}$ vary; *β* is estimated by (21) as *β* ≈ *R*_0_*γ*. Other parameters are as in Table 1. Blue: 0 is the only NE and it is CSNE. Brown: positive *ψ*_*NE*_ < *ψ*_max_ is the only NE and it is CSNE. Light blue: three NEs, 0 and the larger NE are CSNE. Red: maximal feasible vaccination rate is the only CSNE.

To better illustrate how the outcomes depend on different parameter values, Fig 9 shows the NE as *e* and $C_{MPX}^{*}$ vary while *β* = 0.09 (and thus *R*_0_ ≈ 2.11). For these values, there are only two possibilities, either *ψ*_*NE*_ = 0 is the only NE or there is a unique positive NE, 0 < *ψ*_*NE*_ < *ψ*_max_. Fig 10 further illustrates what happens when the transmission rate *β* increases to 0.18, or equivalently, if *R*_0_ increases to 4.23. There are now four distinct regions: the region where 0 is the only NE is almost the same, but the region with unique positive *ψ*_*NE*_ < *ψ*_max_ is significantly smaller and a new region where *ψ*_*NE*_ = *ψ*_max_ appeared for medium values of *e* and large enough values of $C_{MPX}^{*}$. More importantly, there is also a region with three distinct NE. Fig 11 shows the regions of NE when $C_{MPX}^{*}$ and *R*_0_ varies while *e* = 0.85. We can see that the region with three NE exists only for relatively high values of *R*_0_ (about 4 and more). The region exists only for a narrow range of values of $C_{MPX}^{*}$, although the range gets wider as *R*_0_ increases.

**Fig 12:**
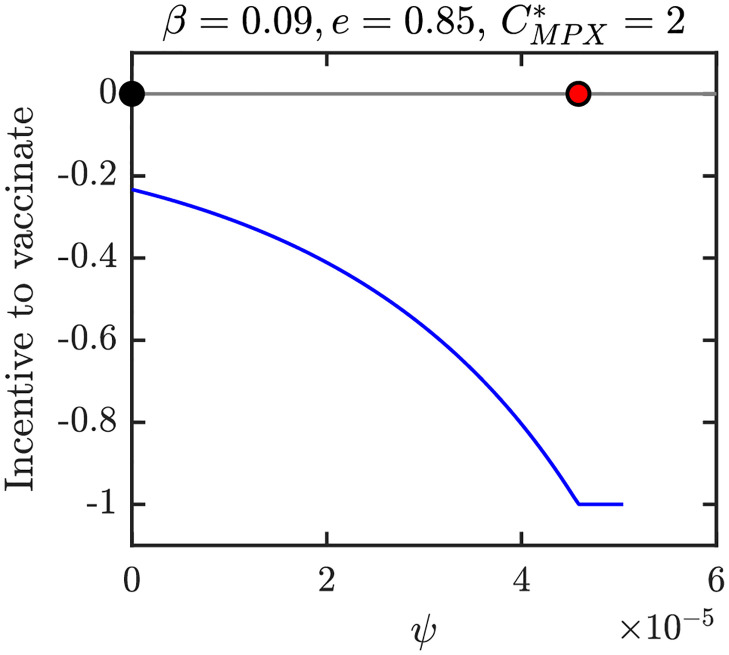
The incentive function for the parameters in the blue region of Fig 9 where 0 is the only NE and CSNE. Full black circle is the CSNE, the red circle corresponds to *ψ*_*HI*_. Unless varied or otherwise specified, the parameters are as in Table 1.

**Fig 13:**
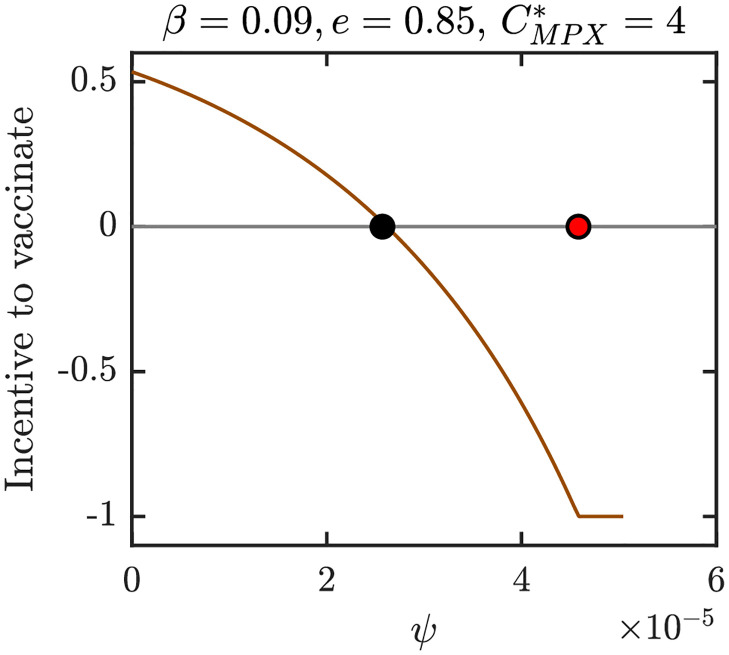
The incentive function for the parameters in the brown region of Fig 9 where *ψ*_*NE*_ > 0 is the only NE and CSNE. Full black circle is the CSNE, the red circle corresponds to *ψ*_*HI*_. Unless varied or otherwise specified, the parameters are as in Table 1.

**Fig 14:**
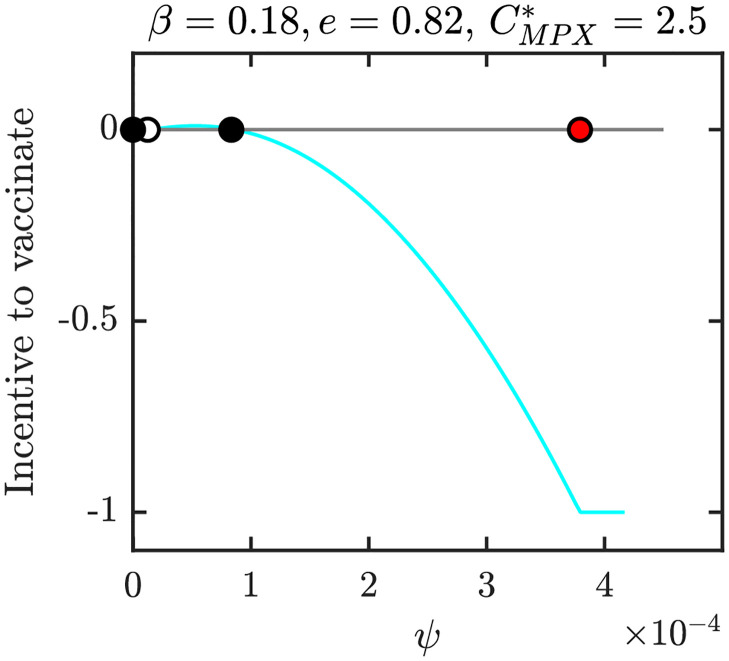
The incentive function for the parameters in the light blue region of Fig 10. There are three NE at the same time. Full black circles are the CSNE. The empty circle is NE that is not CSNE. The red circle corresponds to *ψ*_*HI*_. Unless varied or otherwise specified, the parameters are as in Table 1.

**Fig 15:**
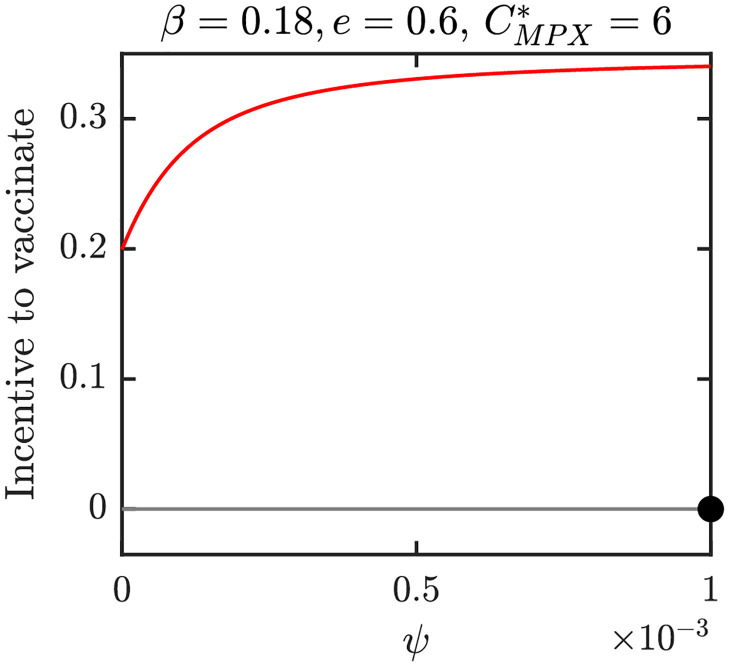
The incentive function for the parameters in the red region of Fig 10. Full black circle is the CSNE. There is no *ψ*_*HI*_. Unless varied or otherwise specified, the parameters are as in Table 1.

Figs 12–15 show graphs of incentive functions corresponding to the different NE regions in Figs 9–11. Specifically, Fig 12 illustrates an incentive function in a region where *ψ*_*NE*_ = 0 is the only NE. Fig 13 is an example of an incentive function where *ψ*_*NE*_ ∈ (0, *ψ*_max_) is the only NE. The incentive function with multiple roots is shown in Fig 14, while the incentive function for the region where NE is the maximal vaccination rate is shown in Fig 15.

**Fig 16:**
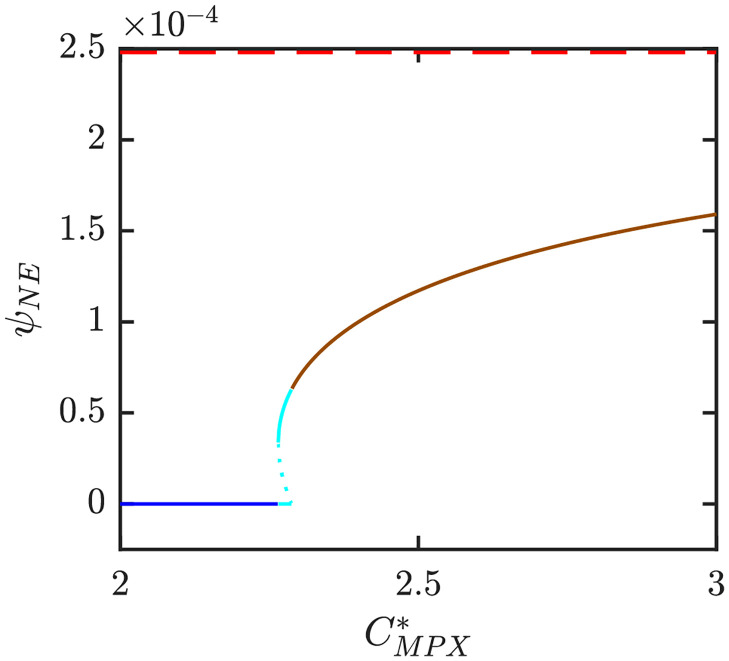
Optimal voluntary vaccination rates when *β* = 0.18. Other parameters are as in Table 1 unless they vary or are otherwise specified. The lines are color coded corresponding to the regions shown in Fig 10 which shows a diagram for Nash equilibria as *e* and $C_{MPX}^{*}$ vary. Blue: 0 is the only NE and it is CSNE. Brown: positive *ψ*_*NE*_ is the only NE and it is CSNE. Red dashed line shows the value of *ψ*_*HI*_. Light blue shows a backward bifurcation when there are three NE at the same time. The full lines are CSNE, the dotted line is not.

**Fig 17:**
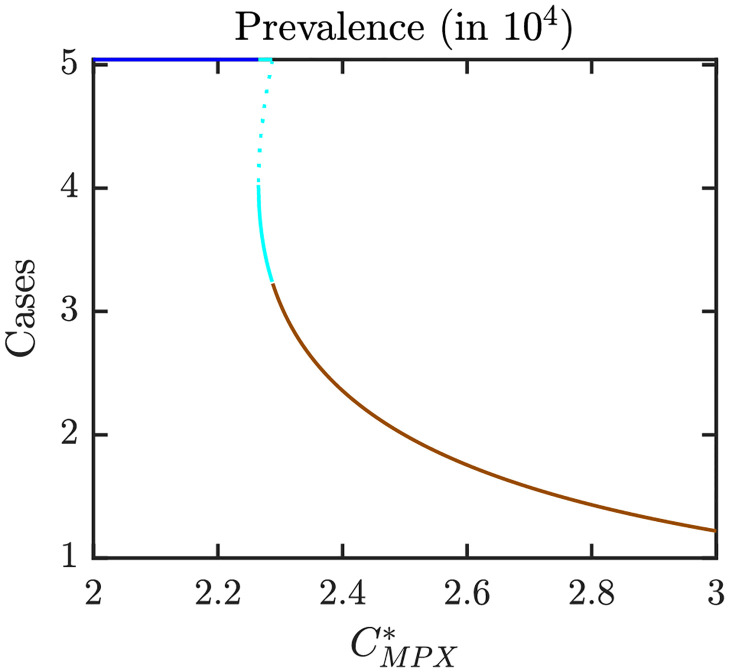
MPX prevalence in a population that uses optimal voluntary vaccination rates; *β* = 0.18 and other parameters as specified in Table 1. The lines are color coded corresponding to the regions shown in Fig 10 which shows a diagram for Nash equilibria as *e* and $C_{MPX}^{*}$ vary. Blue: 0 is the only NE and it is CSNE. Brown: positive *ψ*_*NE*_ is the only NE and it is CSNE. Light blue: three NEs, 0 and the larger NE are CSNE.

**Fig 18:**
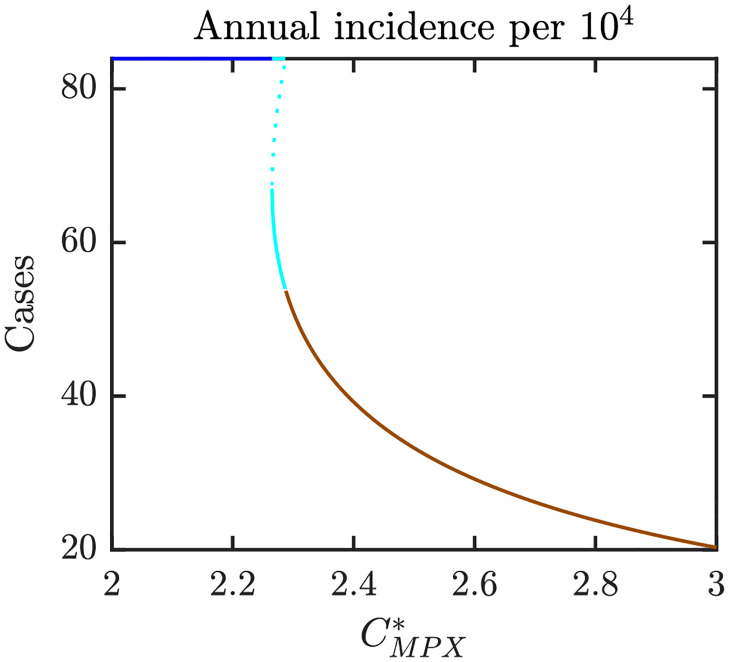
MPX incidence in a population that uses optimal voluntary vaccination rates; *β* = 0.18 and other parameters as specified in Table 1. The lines are color coded corresponding to the regions shown in Fig 10 which shows a diagram for Nash equilibria as *e* and $C_{MPX}^{*}$ vary. Blue: 0 is the only NE and it is CSNE. Brown: positive *ψ*_*NE*_ is the only NE and it is CSNE. Light blue: three NEs, 0 and the larger NE are CSNE.

Figs 16–18 illustrate what happens when the transmission rate *β* increases to 0.18. Without vaccination, the MPX prevalence and annual incidence increase roughly by a factor of 1.5. There is also a backward bifurcation for $C_{MPX}^{*}\approx 2.25$. For those values, we have three optimal voluntary vaccination rates. Only 0 and the largest value are CSNE. The medium vaccination rate, *ψ*_*NE*,1_ is NE but not CSNE. From the public health perspective, it means that there is a need for public policy to mandate the vaccination rate to be at least *ψ*_*NE*,1_; otherwise the voluntary rate would decline to 0. When $C_{MPX}^{*}$ is large enough for *ψ*_*NE*_ > 0 to exist, the vaccination rate is larger in this case compared to case when *β* = 0.09. Consequently, the prevalence and incidence in the population using *ψ*_*NE*_ is lower when *β* is bigger.

**Fig 19:**
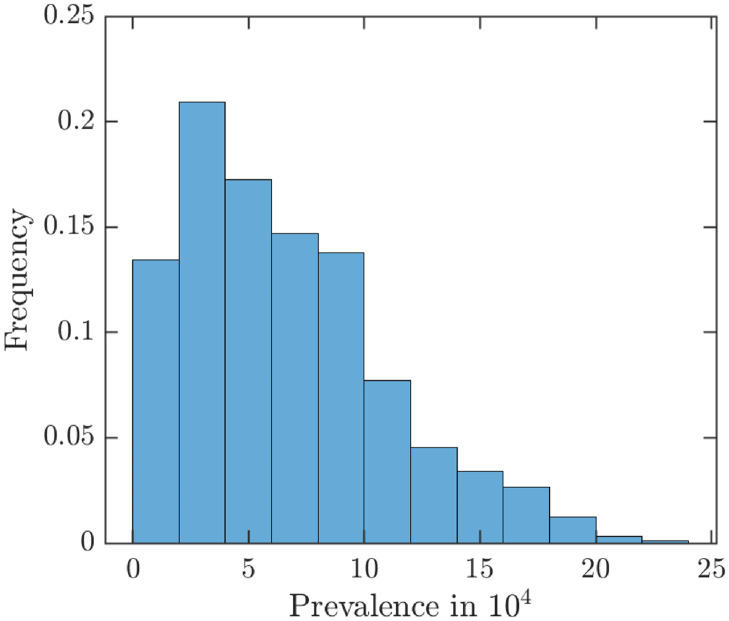
Uncertainty analysis for the MPX prevalence in 10^4^ unvaccinated population (the average is approximately 6.5).

**Fig 20:**
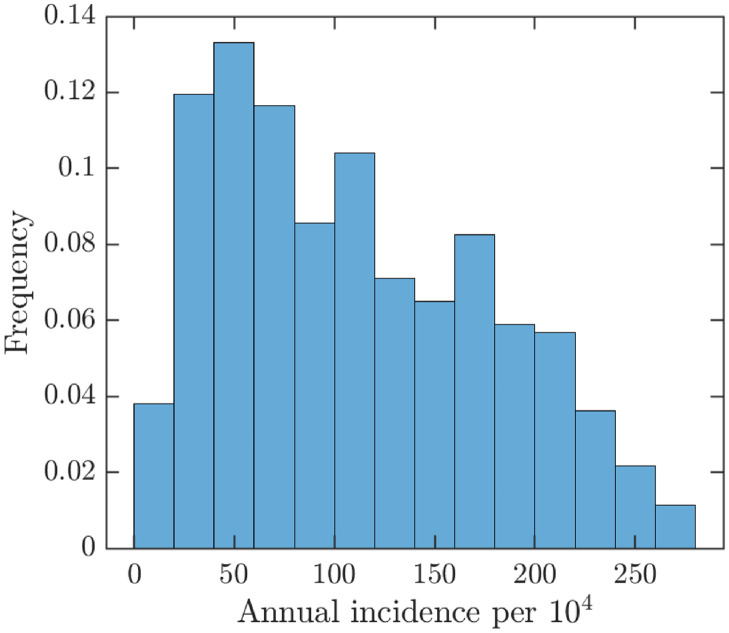
Uncertainty analysis for the MPX annual incidence per 10^4^ unvaccinated population (the average is approximately 110).

To further assess what could happen without vaccination, Figs 19 and 20 show the uncertainty and sensitivity analysis of MPX prevalence and incidence of unvaccinated population. The average prevalence is around 6.5 cases in 10^4^ population and the average annual incidence is around 110 cases per 10^4^ population, further underlying the importance of vaccinations to try to curtail the outbreak.

## 6 Conclusions and discussion

We applied the vaccination game theory framework developed by [71] to the compartmental model of MPX transmission [21] explicitly incorporating the possibility of MPX infections even for the vaccinated population.

Without vaccination, MPX could become endemic with relatively high prevalence (3.5 cases per 10^4^) and incidence (almost 60 cases per year per 10^4^) levels. We identified optimal voluntary vaccinations rates, i.e. rates that are likely to be adopted by the population without any central or government mandates and interventions. For a relatively low cost of MPX infection (less than 2.5 times the cost of the vaccine), to not vaccinate is unfortunately an optimal strategy from the individual standpoint. Even as the cost of infection increases, the optimal voluntary vaccination rate is not enough to substantially decrease the number of MPX cases. Mandatory vaccination for individuals at risk is therefore highly recommended.

The result that voluntary vaccination alone is not enough to eliminate MPX is not surprising. It is a consequence of the low cost of MPX infection (relative to the cost of vaccination). It has already been demonstrated before that the tendency of individuals to optimize self-interest can lead to vaccination levels that are suboptimal for a community [95] and similar predictions have been made in general [96] as well as for specific scenarios involving yellow fever [97], typhoid fever [98], cholera [99], and Hepatitis B [100].

Moreover, we demonstrated that, for some parameter values, specifically a relatively low cost of MPX infection and relatively high rate of MPX transmission, there are multiple Nash equilibria of the vaccination game and the solutions exhibit backward bifurcation. For the public health officials, this means that a minimal vaccination rate has to be mandated in this case, as otherwise the population vaccination rate would decline to 0. The existence of multiple Nash equilibria for vaccination games is a relatively new and not yet fully investigated phenomenon. To our knowledge, [65] is the only other work in the vaccination game theory where multiple equilibria occur for a single action, although the backward bifurcation has not been investigated there; moreover [97] and [101] investigated multiple equilibria in vaccination games with two preventive actions.

Our results underline the importance of proper estimation of the vaccine efficacy and the reproduction number for the current MPX outbreak [102]. The estimates of basic reproduction number *R*_0_ ≈ 2.13 [21], the effective reproduction number *R* ≈ 0.83 [8, 103] and vaccine efficacy *e* ≈ 0.85 [8] are all based on historical data from 1966—1984, during or soon after smallpox vaccinations ended. The current outbreak affects primarily MSM, not children, and there are signs that *R*_0_ can be bigger than expected [104]. If *R*_0_ is larger than 4, then even a slight decrease of vaccine efficacy can mean that even with full vaccination, the MPX outbreak may not be stopped. Moreover, in that scenario, the transmission rate would likely be large enough to have multiple Nash equilibria and backward bifurcation.

As with any other mathematical model, our model has a number of limitations and simplifying assumptions.

We performed the analysis as if MPX already reached the endemic state, which is fortunately not yet the case for most of the countries. The vaccination adoption behaviour can happen at about the same time scale as the infection dynamics, allowing for co-evolution [105]. The coupling of game and epidemic models can lead to oscillations in vaccine uptake over time [95]. The vaccine-generated herd immunity can lower disease incidence so much that real or perceived vaccine risks causes individuals to cease vaccinating which in turn causes uptick in disease incidence [106]. There is even a potential for a significant instability if the perceptions of vaccine and infection risks are homogeneous in the population [95]. We expect that the oscillations would be even more profound with the vaccine imperfection.

Furthermore, we assumed that individuals are well informed about MPX which is also not the case. In the U.S., almost half the respondents (47%) feel that their knowledge level about MPX is poor or very poor [107]. Moreover, infectious diseases can be under-reported, and MPX is no exception with testing only recently expanding [108]. Also, at the time of writing, the demand for the vaccine exceeds the supply [90], i.e. the maximal feasible vaccination rate is relatively low. There is now a growing body of literature on disease transmission and misinformation [109–111]. Misinformation can prevent the suppression of epidemics [112]. It is quite conceivable that, similarly to what happened with HPV vaccine in Denmark [113], a misinformation about MPX vaccine can diminish vaccine coverage.

Perhaps the most severe limitation is that we assumed homogeneous well mixed population and, as a result, we obtained a single Nash equilibrium for most parameter values. Complex networks provide a better platform for more realistic modeling [52, 114–116] and explicitly incorporating social networks within MSM community [117–119] would thus greatly improve the model. Heterogeneity in the population yields the heterogeneity in vaccinating actions [120]. The individuals with many contacts typically have higher inclination to voluntary vaccinate and this can help inhibit the outbreaks [121]. As another extension into heterogeneous populations, one could incorporate the fact that different individuals can perceive the cost of MPX and the cost of vaccination differently. This assumption could significantly alter our results. In the present (homogeneous) model, if the cost of MPX is smaller than the cost of vaccination, individuals opt not to vaccinate. However, in the heterogeneous model, even if the average cost of MPX is smaller than the average cost of vaccination, there can still be a non-negligible proportion of population who perceive the cost of MPX as significantly larger than the cost of vaccine and, as a result, opt to vaccinate.

One has to keep in mind that no model can be fully realistic and account for every detail [122, 123] and so, despite all the above shortcomings, our model provides a reasonable, and not so positive, outlook into what could happen without any mandate for vaccinations and/or possibly other measures to stop the unfolding MPX outbreak.

## Supporting information

S1 Code Matlab code used for generating the figures. (TXT)

S2 Code SAS code used for generating the incidence rates. (TXT)
